# Taking a Promising Vaccine Candidate Further: Efficacy of ASFV-G-ΔMGF after Intramuscular Vaccination of Domestic Pigs and Oral Vaccination of Wild Boar

**DOI:** 10.3390/pathogens11090996

**Published:** 2022-08-31

**Authors:** Paul Deutschmann, Tessa Carrau, Julia Sehl-Ewert, Jan Hendrik Forth, Elisenda Viaplana, Jose Carlos Mancera, Alicia Urniza, Martin Beer, Sandra Blome

**Affiliations:** 1 Institute of Diagnostic Virology, Friedrich-Loeffler-Institut, Suedufer 10, Insel Riems, 17493 Greifswald, Germany; 2 Department of Experimental Animal Facilities and Biorisk Management, Friedrich-Loeffler-Institut, Suedufer 10, Insel Riems, 17493 Greifswald, Germany; 3 Zoetis Manufacturing and Research Spain, Finca la Riba, Carretera de Camprodon s/n, L’Hostalnou de Bianya, 17813 Girona, Spain; 4 Zoetis Belgium, Mercuriusstraat 20, 1930 Zaventem, Belgium

**Keywords:** African swine fever, vaccination, efficacy, domestic pigs, wild boar, oral vaccine, intramuscular vaccine

## Abstract

African swine fever (ASF) is a pandemic threat to the global pig industry and wild suids. A safe and efficacious vaccine could monumentally assist in disease eradication. In the past years, promising live attenuated vaccine candidates emerged in proof-of-concept experiments, among which was “ASFV-G-∆MGF”. In our study, we tested the vaccine candidate in three animal experiments intramuscularly in domestic pigs and orally in wild boar. Further, a macrophage-grown vaccine virus and a virus grown on permanent cells could be employed. Irrespective of the production system of the vaccine virus, a two-dose intramuscular immunization could induce close-to-sterile immunity with full clinical protection against challenge infection. After oral immunization, 50% of the vaccinees seroconverted and all responders were completely protected against subsequent challenge. All nonresponders developed ASF upon challenge with two acute lethal infections and two mild and transient courses. The latter results show a lower efficiency after oral administration that would have to be taken into consideration when designing vaccination-based control measures. Overall, our findings confirm that “ASFV-G-∆MGF” is a most promising vaccine candidate that could find its way into well-organized and controlled immunization campaigns. Further research is needed to characterize safety aspects and define possible improvements of oral efficiency.

## 1. Introduction

African swine fever (ASF), caused by the African swine fever virus (ASFV), is a notifiable disease of pigs that has become a tangible pandemic threat to domestic and wild pigs [1]. Currently, more than 35 countries in five world regions (Africa, Asia, Europe, Oceania, and the Americas) are affected and the disease continues to spread (OIE situation report, visited online at African swine fever OIE—World Organisation for Animal Health; 23 April 2022). There is presently no licensed vaccine or treatment option for the disease, which can present with the clinical signs of a viral haemorrhagic fever and very high lethality [2].

While the classical veterinary hygiene measures, i.e., culling of affected farms, establishment of restriction zones and movement bans, can be successfully implemented for industrial pig farms, a spread of the disease in regions with small, family-owned farms and lack of financial compensation for losses can hardly be stopped with the possibilities available so far [3]. The same applies to outbreaks of ASF in wild boar populations, especially if the disease is introduced over a broader front rather than punctually. 

The challenges we are facing in the context of the current ASFV pandemic are difficult to solve without the help of a vaccine, and thus a safe and efficacious vaccine for both parenteral and oral bait application could provide the additional tool that is still missing to regain the upper hand over the disease [4]. Among the most promising ASF vaccine candidates that have shown high potential in pilot studies are genetically engineered live attenuated vaccines, i.e., deletion mutants created through homologous recombination [5,6,7]. One of the first reported vaccine viruses with rational deletions was “ASFV-G-∆9GL” [8]. This virus showed residual virulence that could be reduced by an additional deletion of the UK gene [9]. Another deletion mutant that was investigated in more detail was “BA71∆CD2”. This vaccine candidate was able to induce strong humoral and cellular responses, conferred solid protection against the homologous virus (BA71) in a dose-dependent manner, and held some promise for cross-protection [10]. Another mutant virus with deletions of the CD2 and UK genes offered full protection against homologous challenge, but allowed residual replication of the challenge virus [11]. Recently, “ASFV-G-∆A137R” was reported to induce full protection against homologous challenge, but the vaccine-virus-induced viremia was medium to high [12]. Close-to-sterile protection against homologous challenge, and at the same time, low-to-moderate vaccine-virus-induced viremia were achieved with “ASFV-G-∆I177L” [13] and “ASFV-G-∆MGF” [14]. The latter of these particularly promising recombinant vaccines harbors deletions in the multigene families 360 and 505. The same modifications in the MGF regions with additional deletion in the CD2 gene from a Chinese backbone virus also offered full attenuation and protection [15].

Here, we analyzed “ASFV-G-∆MGF” further and investigated its efficacy in three independent animal trials. Two trials were designed to assess safety and efficacy of a double intramuscular vaccination scheme of domestic pigs. In the first trial, a macrophage-grown virus was used, while in the second, the vaccine virus master seed was grown on a commercial permanent cell line (subject to patent restrictions). The third trial was performed as a proof-of-concept study with a single oral immunization of wild boar using the cell-culture-grown vaccine virus. Oronasal challenge infection of the domestic animals was performed with the highly virulent ASFV strain “Armenia 2008”; the wild boar trial included an oronasal challenge infection with the recent ASFV strain “Germany 2020”. All animals were monitored for clinical signs and were investigated using accredited routine virological and serological methods.

## 2. Results

### 2.1. Clinical Signs and Pathological Lesions

#### 2.1.1. Domestic Pig Trial A 

##### “ASFV-G-∆MGF”, Intramuscular

**Figure 1:**
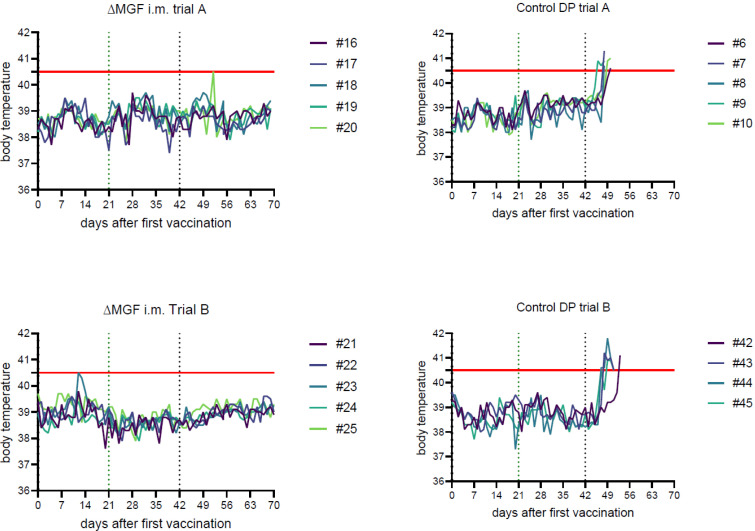
Daily body temperatures of animals recorded throughout the course of each respective animal trail. Individuals are depicted in different colors. The red line marks the threshold for fever at 40.5 °C. The green dotted line marks boost vaccination at 21 dpv; the black dotted line marks the challenge infection at 42 dpv.

The five vaccinees remained completely healthy after both immunizations and after challenge infection, with only one deviation observed. Pig #20 had a febrile body temperature of 40.5 °C on 9 days post-challenge (dpc); however, it remained free of clinical signs and recovered the next day (see Figure 1). No pathomorphological abnormalities were found except for mild pulmonary consolidation in pig #17 and a variable dark reddening of renal and hepatogastric lymph nodes.

##### Challenge Controls

Following infection with ASFV “Armenia08”, the five control animals developed fever up to 41.3 °C (see Figure 1), accompanied by anorexia, reduced liveliness, and reddening of the skin. Animals were euthanized according to our moderate humane endpoint between 6 and 8 dpc with a maximum clinical score (CS) of 5.5. Typical lesions associated with an ASFV infection were identified during necropsy: severely enlarged haemorrhagic lymph nodes in all animals, extensive gallbladder wall edema and marked alveolar pulmonary edema (#8, #9). Myo- and endocardial haemorrhages were observed in pig #8. Mild ascites and kidney haemorrhages were present in animal #9.

#### 2.1.2. Domestic Pig Trial B

##### “ASFV-G-∆MGF”, Intramuscular

All five animals remained healthy. Only one pig (#23) displayed an elevated body temperature of 40.5 °C on 12 days post-vaccination (dpv) (see Figure 1) without any other clinical abnormalities. No other clinical signs or febrile temperatures were observed after immunizations or challenge infection. Postmortem examination revealed no macroscopic abnormalities except for variable dark reddening of tracheobronchial, renal, and hepatogastric lymph nodes in animals #22, #23, and #24.

##### Challenge Controls

The four control pigs displayed fever up to 41.8 °C, clinically reflected by anorexia and reduced liveliness, and were euthanized between 8 and 11 dpc at a maximum CS of 5. Gross lesions included haemorrhagic enlarged lymph nodes in all animals, petechiae on kidneys and / or urinary bladder (#43, #45), and in some cases pulmonary consolidation (#43, #44). One control animal (#45) had developed severe haemoperitoneum.

#### 2.1.3. Wild Boar Trial

##### “ASFV-G-∆MGF”, Oral

All eight wild boar remained healthy after immunization, and no clinical signs were observed. Following challenge infection, animals #62 and #67 displayed reduced appetite and liveliness beginning at 5 dpc. Clinical signs worsened during the following days and were eventually accompanied by vomitus and reddened skin and eyes. On 8 dpc, #62 reached a not-yet-critical CS of 6.5, but died during the day in a peracute convulsive seizure within minutes, before the animal could be released by euthanasia. Animal #67 reached the humane endpoint with 12 cumulative CS points and was euthanized at 9 dpc, when it showed severe apathy and anorexia, labored breathing, and reddened ears. Wild boars #61, 63, and 65 developed slightly reduced appetite and liveliness between 5 and 9 dpi (maximum CS of 2); however, they recovered thereafter. Animal #61 reached one CS point for reduced liveliness again on 13 dpc, but was clinically inapparent thereafter. #64 and 68 did not show clinical abnormalities. Macroscopically, in animals #62 and 67, which died or were euthanized, mild-to-severe haemorrhages were present in the kidneys, in various lymph nodes and in the gastrointestinal tract. Serosanguinous peritoneal and thoracic effusion was present in animals #62 and 67, respectively. In addition, severe alveolar edema was found in animal #62, whereas animal #67 revealed an enlarged, friable spleen and pinpoint haemorrhages in the urinary bladder. In contrast, wild boar #61, 63, 64, 65, 66, and 68 mainly showed very mild lesions, including reddening of the hepatogastric and renal lymph nodes. Animals #61, 64, and 65, moreover had mild-to-moderate enlarged friable spleens. Focal mild pulmonary consolidation was observed in pigs #63 and 64. Multifocal pinpoint haemorrhages were detected in the lung of animal #66. 

##### Control Group

The four control WB showed an onset of reduced liveliness and apathy at 4 or 5 dpc, worsening and reaching the humane endpoints at 7 dpc, when they were completely anorectic and apathic (cumulative CS between 4 and 7). Animals #72 and 69 showed reddening of the skin around the ears. Labored breathing was observed in animals #69 and 71. 

All pigs revealed multifocal pinpoint haemorrhages in the kidney and large intestine. Up-to-severe haemorrhages were observed in multiple lymph nodes. All pigs except #69 showed haemorrhagic and necrotic areas in the liver. Mild haemorrhages of the urinary bladder and diffuse pancreatic necrosis were observed in #70 and 72, respectively.

### 2.2. Genetic Characterization of the Vaccine Virus

Next-generation sequencing of the vaccine virus grown on the permanent cell line yielded 1.7 M 150 bp ASFV reads, resulting in a full genome with an average depth of 1394 per nucleotide. In comparison to the original “ASFV-G-∆MGF” sequence, only two point mutations were found: one silent in the B438L gene at position 98378 (A🡪G) and one in B438L at position 98378 (C🡪G), which leads to an amino acid replacement alanine 🡪 proline with unknown consequences.

### 2.3. ASFV Genome Detection

#### 2.3.1. Domestic Pig Trial A

##### “ASFV-G-∆MGF”, Intramuscular

Two individual blood samples (animals #18 and #19, respectively) were positive prior to challenge infection on 7 and 21 dpv, (3 and 200 gc, see Table 1). After challenge infection, traces of ASFV genomes were detected in animal #16 on 4 dpc, and in animal #20 at 10 and 14 dpc (<1 genome copy (gc)). All other samples were PCR-negative. One blood sample taken after challenge infection was positive for challenge virus genomes in the differentiating PCR (#10, 10 dpc); the other samples with only traces of genome were not detected in the gel-based PCR system. Swab samples taken throughout the trial were negative for ASFV genomes (see Supplementary Table S3).

All organ samples taken from animals #16 to #19 were negative in the qPCR. Weak positive results were obtained for animals #20 with 10 gc detected in the popliteal lymph node and viral genome traces in a lung sample (see Table 2).

##### Challenge Control

The first genome-positive samples appeared matching with the onset of fever on 4 dpc (see Figure 1 and Table 1). Upon necropsy (humane endpoints between 6 and 8 dpc), all control animals were positive for ASFV genomes in tissues and blood samples (up to 1.1 × 10^6^ gc, see Table 2 and Supplementary Table S2).

#### 2.3.2. Domestic Pig Trial B

##### “ASFV-G-∆MGF”, Intramuscular

Upon immunization, two individual blood samples gave weak positive results in qPCR on 7 and 14 dpv, respectively (<4 gc, see Table 1). After challenge infection and until the end of the trial at 28 dpc, all blood and swab samples remained negative for ASFV genomes. The complete panel of tissue samples was negative for ASFV genomes (see Table 2).

##### Challenge Control

Positive results from control animals in qPCR and virus isolation emerged a short time before the onset of fever as early as 4 dpc (see Figure 1 and Table 1). At day 7 pc, all pigs were positive in blood and swabs for ASFV genomes. When the humane endpoint was reached at 8 to 11 dpc, all blood and tissue samples (see Table 1 and Table 2) were positive for viral genomes (up to 1.5 × 10^5^ gc).

#### 2.3.3. Wild Boar Trial

##### “ASFV-G-∆MGF”, Oral

At 21 dpv, viral genomes were detected in the blood of animals #61, 63, and 65 (<96 gc, see Table 1); the other animals were negative. At necropsy, animals #62 and 67 were highly ASFV genome-positive in all tissues and blood (up to 10^5^ gc). Traces of genomes (<10 gc) were found in some organs of #61, 66, and 64 (see Table 2). These corresponding qPCR-positive samples of #61, 66, and 64 were tested in gel-based differentiating PCR, but no ASFV genomes could be detected in this system. Animals #63, 65, and 68 were qPCR-negative in the complete panel of samples taken at necropsy.

##### Controls

All control animals were highly positive for ASFV genomes in the complete sample set upon necropsy (up to 4 × 10^5^ gc, see Table 1 and Table 2).

### 2.4. Detection of ASFV-Specific Antibodies

#### 2.4.1. Domestic Pig Trial A

**Figure 2:**
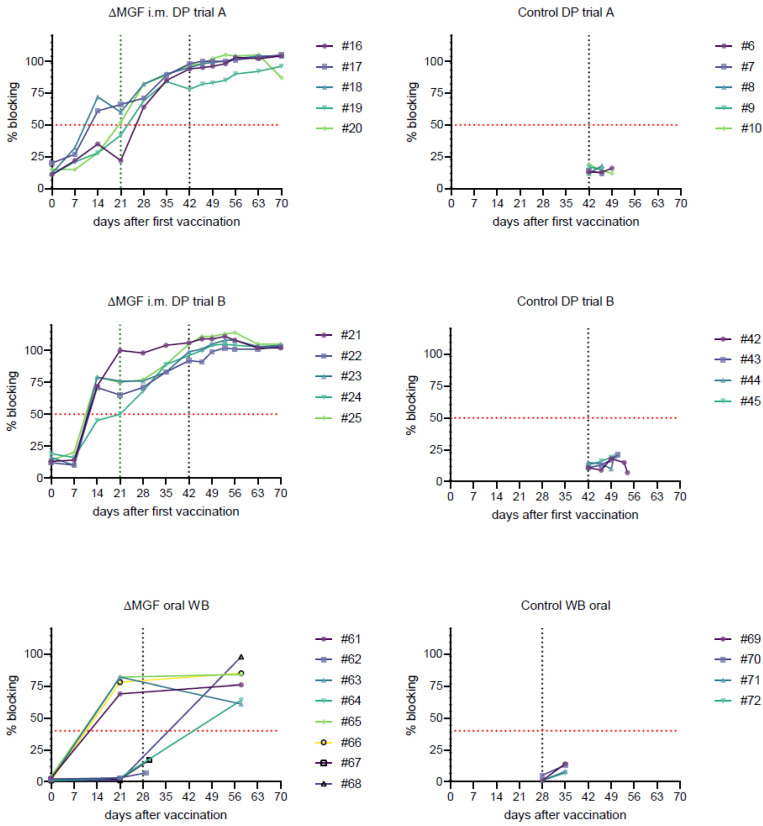
Percentual blocking in the ELISA systems deployed for samples from the respective vaccine trials after vaccinations and challenge infection. Red dotted line marks the threshold for positivity. Individuals are depicted in different colors. The green dotted line marks boost vaccination at 21 dpv (DP/domestic pigs); the black dotted line marks the challenge infection at 42 dpv (DP) or 28 dpv (wild boar/WB), respectively.

The first pigs were positive in the p72 antibody ELISA in the “ASFV-G-∆MGF”-vaccinated group on 14 dpv, with four out of five animals giving positive results and one animal having a doubtful ASF antibody result on 21 dpv. All animals remained positive from 28 dpv onwards. Control pigs were negative for ASFV specific antibodies throughout the trial (see Figure 2).

#### 2.4.2. Domestic Pig Trial B

Four out of five animals were positive at 14 dpv, along with one doubtful result. From 21 dpv until the end of the trial at 28 dpc, all animals remained positive. The controls remained negative (see Figure 2).

#### 2.4.3. Wild Boar Trial

Four out of eight vaccinated WB were positive in the p32, p64, and p72 specific antibody ELISA on 21 dpv (#61, 63, 65, and 66); the other animals remained seronegative before challenge infection (see Figure 2). Animals #62 and 67 were still negative when they died/were euthanized. Animals #64 and 68 had seroconverted until 28 dpc. The seronegative status of #64 and 68 at 21 dpv was confirmed by additionally testing the samples in the INGEZIM PPA COMPAC ELISA and in highly sensitive IPT (data not further shown).

## 3. Discussion

With the pandemic spread of ASFV, vaccine research was intensified and promising candidates emerged. Looking at proof-of-concept data of fully efficacious vaccine candidates, one may assume that a licensable vaccine might be closer than it appears [7]. The type of vaccine that is needed may differ depending on the affected region and the disease scenario. While a vaccine for use in domestic pigs could be suitable for epidemiolocal scenarios with frequent introductions into domestic pig backyard holdings in Europe, Africa, or Asia, for central Europe, the epidemiological situation focuses on wild boar. Vaccination of free-ranging wildlife calls for a bait-based formulation; consequently, an oral vaccine is needed here, similar to the successfully used preparation against classical swine fever [16]. Oral vaccine formulations require a live vaccine approach, because only replicative virus is taken up through intact mucosa. In the end, both intramuscular and oral vaccine-delivery options are conceivable for different field-use scenarios for an ASFV vaccine candidate.

Consequently, we took one of the most promising candidates, “ASFV-G-∆MGF” [14], further confirmed the previously shown full efficacy for intramuscular vaccination, and assessed the possibility of using the vaccine candidate for oral administration. Going beyond this aspect, we examined effects of passaging of the vaccine virus on a permanent cell line. 

In both domestic pigs and wild boar, the prototype vaccine was completely attenuated and innocuous and did not cause any traceable harm to the animals. No differences in efficacy or attenuation between the macrophage-derived vaccine and the vaccine derived from a permanent cell line were observed in either domestic pig study. Genetic characterization of the permanent cell-line-derived vaccine yielded only two point mutations, one silent and one without known consequences. Differences in vaccination efficiency, i.e., induction of vaccine virus replication and subsequent host responses, between the oral and the intramuscular application were apparent, however. These differences reflect the situation with field virus strains where oral infection is much less efficient [17].

In the intramuscularly immunized domestic pigs, full clinical protection was observed in all vaccinees and no challenge virus replication was detectable in 9/10 pigs. Only in a single animal were traces of challenge virus replication observed, and macroscopic lesions were largely absent in both groups. Traces of vaccine virus replication were observed in 4/10 animals. The reddening of lymph nodes, which were present in 7 out of 10 animals, most likely indicates previous virus-induced haemorrhage in the lymph node itself or drainage of a bleeding in the tributary area [18,19]. Our data suggest that both vaccine and challenge virus were eliminated by the end of both trials, as not even traces of viral genome were found in any tissues or blood in nine out of ten animals. Thus, vaccine candidate “ASFV-G-∆MGF” showed a reproducible efficacy after intramuscular immunization against challenge infection with a homologous ASFV strain of genotype II. Since the results in our harmonized experimental setup between DP trial A and B are very similar, the minor genetic adaptions that were found in the permanent cell-line-derived vaccine are likely without any consequences for vaccine safety and efficacy. With our study design comprising two vaccinations, we exceeded the already promising results achieved by O’Donnell and Holinka [14] in their experimental setup with a single vaccination. Unlike in the preceding study, we observed no febrile reactions with clear correlation to vaccination or challenge infection and detection rate of both vaccine and challenge virus in blood samples was much lower in our study. Comparison of our results suggests that while a single dose of immunization is sufficient to achieve clinical protection by intramuscular immunization, adding a boost immunization could reduce viremia, thereby contributing to optimized safety prospects of the vaccine candidate. 

In the wild boar orally immunized with “ASFV-G-∆MGF”, all responders were protected, but not all animals responded to oral immunization, as evidenced by the lack of seroconversion. In detail, only 50% of the animals had seroconverted after 21 dpi. These animals specifically showed a high level of protection after challenge infection. Only transiently reduced liveliness and appetite were observed in animals #61, 63, and 65 (maximum of two cumulative cs points). Seronegative animals #62 and 67, however, displayed signs of severe disease and died or were euthanized. At necropsy, both animals showed characteristic lesions for ASF. 

Interestingly, seronegative animals #64 and 68 survived challenge infection without displaying obvious clinical signs, but both had seroconverted upon necropsy. This could be due to biological variability, slight attenuation of the German field ASFV strain used in this trial, or vaccine-induced protection. While the presence of antibodies cannot be seen as a correlate for protection, the authors know of no ASFV vaccine candidate to induce protection without seroconversion, and thus, vaccine-induced protection is rather unlikely. In this pilot trial, other correlates could not be evaluated (e.g., T-cell or interferon responses). Principal functionality of our challenge model can be shown by the fast and severe onset of ASFV in the control animals, who received the identical virus by the same route. Still, oronasal application does hold certain insecurities due to possible differences in virus uptake. The animal’s behavior, individual levels of proteases in the saliva, and the susceptibility of the mucosa influences the efficacy and renders the system more error-prone [17,20]. 

It seems likely that variability in virulence phenotype of the German ASFV field strain led to the observed differences. In conclusion, the experiment has shown that a single oral dose of “ASFV-G-∆MGF” can induce full protection against field virus challenge in at least 50% of vaccinated animals. Compared to the recently evaluated vaccine candidate “ASFV Lv17/WB/Rie1” that was reported to induce antibodies in 10/12 animals and protection against lethal infection in 11/12 animals [21], the vaccination efficiency of “ASFV-G-∆MGF” was lower. However, while vaccine virus shedding and chronic lesions could be observed for the non-haemadsorbing candidate “ASFV Lv17/WB/Rie1” [22], indications for a chronic disease course, which would be a major safety concern, were not observed for the “ASFV-G-∆MGF” vaccine candidate. 

While a higher efficiency of oral immunization would be desirable for the latter, the pandemic situation may not allow us to wait for the perfect vaccine and benefit–risk analyses are needed, but chronic disease courses caused by vaccines must be ruled out. Improvement of efficiency for “ASFV-G-∆MGF” may be reached if more than one bait dose is taken up by the animals, an event that may be inevitable in a natural application when animals have repetitive access to baits, as in the classical swine fever example [23]. Moreover, virus delivery could offer opportunities of optimization. A conceivable approach could be a more viscous medium in the bait to delay swallowing of the virus suspension, preventing quick inactivation in the stomach. 

To date, there is only one report of another ASFV prototype vaccine of the same kind where full protection was achieved against lethal challenge by oronasal immunization with “ASFV-G-ΔI177L” [13,24]. Here, all animals were protected; however, the inoculation route differs. Oronasal inoculation offers the vaccine virus suspension an increased contact surface to mucosae, possibly enhancing virus uptake. While the success reported here is exciting, it remains a rather artificial immunization route, since wildlife will most likely take up baits by feeding on them, only offering contact to the oral mucosa. 

Here, we report that “ASFV-G-∆MGF” is genetically stable after cultivation on a permanent cell line, a major benefit for future commercialization, as it can allow production of large quantities of vaccine virus. The same was recently shown for “ASFV-G-ΔI177L” [25], underlining that both vaccine candidates are highly auspicious. 

There are additional prototype vaccines that successfully protect animals intramuscularly. One example is genetically similar candidate “HLJ/18-7GD” [15]. This deletion mutant, based on modifications in the same six MGF-genes plus the extra deletion in the CD2v gene, could also induce a full clinical protection after challenge infection with the virulent backbone strain “HLJ/18“. Little to no residual viral replication was detected and complete attenuation in pigs was observed. While direct comparison is hindered by different experimental setups, both candidates showed an equally good intramuscular efficacy, and an oral vaccination study could be auspicious for this candidate.

In order to bring any vaccine closer to licensing, now that we have identified a few efficacious prototypes, more insight into safety characteristics is needed, especially with the prospect of releasing infectious vaccine viruses in the field. 

For “ASFV-G-ΔMGF”, we have proven a measurable replication in the pig, although to a rather limited extent and with no detection of virus shedding. Additional safety trials are needed as a basis for thorough benefit–risk analyses. For this, research and legal authorities should now work together to define the most relevant knowledge gaps and to concentrate further research on these urgent open questions. In the EU, European Pharmacopoeia defines clear requirements for vaccines, and further studies should specifically address these regulatory aspects to speed up the search for a candidate that is suitable for licensing and could thus eventually be available for use.

## 4. Materials and Methods

### 4.1. Experimental Settings and Animals

The complete study comprised three animal experiments, domestic pig (DP) trials A and B, and a wild boar (WB) trial. Domestic pigs were 6–8-week-old crossbred animals bought from the same commercial farm, but from different groups and born approximately 5 months apart. The wild boar enrolled in the study were approximately 6 months old and obtained from two game parks in Brandenburg and Mecklenburg–Western Pomerania, Germany. In DP trial A, 5 vaccinees (numbered #16–20, see Table 2) and 5 control animals (#6–10) were kept; DP trial B consisted of 5 vaccinees (#21–25) and 4 controls (#42–45); and the WB experiment comprised 8 vaccinees (#61–68) and 4 controls (#69–72). All animals were randomly allocated to groups. All animals were moved to the high-containment facilities of the Friedrich-Loeffler-Institut (FLI) and were kept under appropriate containment and animal welfare conditions. Upon arrival, individuals were ear-tagged and the absence of ASFV-related antibodies and genome was confirmed at the start of each trial. Pigs and wild boar were fed a commercial pig feed appropriate for their age, mixed with hay cobs, and had ad libitum access to water. A sufficient acclimatization phase was ensured before the start of each trial.

In both DP trials, animals received two intramuscular vaccinations with 1 mL of virus suspension, respectively. A dose of roughly 10^4^ HAD_50_ was administered at both vaccinations in DP trial A and roughly 10^3^ HAD_50_ in DP trial B (see Supplementary Table S1 for back titrations). Boost was performed 21 days after the first dose. Challenge infection followed on 42 dpv. Immunizations were administered deep into the muscle of the right neck with a 2 mL syringe with 20 G cannula. 

The WB received 2 mL of virus solution orally at 10^5^ HAD_50_ with a 5 mL syringe, placed on top of the tongue (see Supplementary Table S1 for back titrations). Challenge infection was conducted at 28 dpv. Oronasal application of the challenge virus suspension was conducted by delivering 0.5 mL into each nasal orifice and 1 mL into the oral cavity using a 3 mL syringe for both the DP and WB trial. All animals were monitored for 28 dpc.

Upon first vaccination until the end of trial, clinical parameters were monitored as previously described [26]. Further, the rectal body temperature of each domestic pig was recorded daily. For the WB, temperature recording was not possible due to the need for immobilization for such procedures. Fever was defined as a body temperature above 40.5° C. Clinical parameters were liveliness, skin alterations, posture, ocular irritations, breathing, gait, feed intake, and defecation. They were assigned to points according to the severity of findings with a range between 0 (asymptomatic) and 3 points (severe). The sum of points was recorded as a cumulative CS, and under consideration of body temperatures, used to define humane endpoints. A moderate humane endpoint was applied in both trials at a CS of ≥10 points or in case of unjustifiable sufferings according to the assessment of the responsible veterinarian. Moreover, domestic pigs had to be put down when they displayed fever for three consecutive days accompanied by any other clinical sign, or for four days without accompaniment of other clinical signs.

During the trials, levels of viremia and serological parameters, as well as shedding for the DPs, were investigated. For this purpose, DPs were sampled on 0, 7, 14, 21, 28, 35, and 42 dpv, and on 4, 7, 10, 14, and 21 dpc, collecting EDTA blood and native blood for serum preparation from the jugular vein along with deep oropharyngeal swabs. For the WB, sampling was reduced due to the high susceptibility to stress and the need for immobilization before handling. They were sampled on 0 and 21 dpv (vaccinees) and 0 dpc (controls) as well as upon necropsy. 

When animals reached the humane endpoint or the end of the trial, they were put in deep anaesthesia with a combination of tiletamine/zolazepam (Zoletil^®^, Virbac, Carros, France), xylazine (Xylazin 20 mg/mL, Serumwerk Bernburg, Bernburg, Germany) and ketamine (Ketamin 10%, medistar, Houston, TX, USA) and killed by exsanguination.

All animals underwent full necropsy and were macroscopically scored based on a standardized protocol [27] with slight modifications. EDTA blood and native blood for serum preparation were collected in addition to a panel of organ samples (see Table 2).

Compliance with EU Directive 2010/63/EC and institutional guidelines was assured. Trials were approved by the competent authority (Landesamt für Landwirtschaft, Lebensmittelsicherheit und Fischerei Mecklenburg-Vorpommern) under reference numbers LALLF 7221.3-1.1-003/20 and -035-21).

### 4.2. Cells

In the framework of the reported trials, virus cultivation, re-isolation, and titrations were conducted on peripheral blood mononuclear cell (PBMC)-derived macrophages. PBMCs were produced from blood of a healthy donor pig as previously described [20].

### 4.3. Vaccine and Challenge Viruses

#### 4.3.1. Vaccine viruses

The vaccine virus master seed “ASFV-G-∆MGF” [14] was provided by Zoetis Manufacturing & Research; S.L. Virus in DP trial A originated from passage in PBMC-derived macrophages. For DP trial B and the WB trial, virus was passaged once in a commercial permanent cell line (subject to patent restrictions) and provided ready-to-use by Zoetis. The virus originating from a permanent cell line was characterized by next-generation sequencing to reveal possible genetic modifications inflicted by cell-culture passage. To this means, DNA was sent to and sequenced by Eurofins Genomics. This service included preparation of a 450 bp DNA sequencing library using a modified version of the NEBNext Ultra™ II FS DNA Library Prep Kit for Illumina and sequencing on an Illumina NovaSeq 6000 with S4 flowcell, XP workflow and in PE150 mode (Illumina, San Diego, CA, USA).

#### 4.3.2. Challenge Virus 

The highly virulent ASFV “Armenia 2008” strain used for the DP trials was obtained from the German National Reference Laboratory (NRL) for ASF (FLI, Insel Riems, Germany) and was administered to the animals as macrophage cell-culture supernatant. Like the mutual backbone virus of the deletion mutants in this trial, this well-characterized strain belongs to genotype II and represents field strains of the current epidemic. It shares almost 100% identity with “Georgia07” and is also highly virulent [28].

The challenge virus for the WB trial, ASFV “Germany 2020” (genotype II, German variant IV, ASFV/GER/2020/WB/IV_SN) was isolated from a wild boar carcass found in Saxony, Germany, in 2020. It was previously characterized at the NRL and showed high virulence in domestic pigs. The strain that shares >99% identity with ASFV “Armenia 2008” was chosen to represent the current situation in the field. The virus was passaged once in domestic pigs in a preceding animal trial at the NRL. It was administered as sea sand homogenate of infected spleen tissue in RPMI-1640 cell-culture medium. Viruses used for challenge infections were back-titrated to roughly 10^5^ HAD_50_ (DP trial B) or 10^4^ HAD_50_ (DP trial A and WB trial, see Supplementary Table S1).

### 4.4. Laboratory Investigations 

#### 4.4.1. Processing of Samples

Serum was obtained from native blood through centrifugation at 2500× *g* for 15 min at 20 °C. Swabs were soaked in 1 mL of RPMI-1640 cell-culture medium at 20 °C for one hour, then vortexed thoroughly and aliquoted. Tissue samples were homogenized for nucleic acid extraction with a metal bead in 1 mL phosphate-buffered saline (PBS) at 30 Hz for 3 min using a TissueLyser II (Qiagen). All samples were stored at −80 °C until further use.

#### 4.4.2. Virus Detection

For qPCR, viral nucleic acids were extracted from blood and tissue samples using the NucleoMag Vet Kit (Machery-Nagel) on the KingFisher^®^ extraction platform (Thermo Scientific) or the manual QIAamp^®^ RNA Viral Mini Kit (Qiagen. The qPCR was conducted employing the protocol published by King et al. [29] or with a commercial qPCR (virotype 2.0 ASFV, Indical Bioscience, Leipzig, Germany). All PCRs were performed on C1000™ thermal cyclers with the CFX96™ Real-Time System (Biorad). Results were recorded as quantification cycle (cq) and genome copy (gc) values, calculated by applying an ASFV in-house full-genome standard. For differentiation between vaccine and challenge viruses, tailored PCR targeting a deletion site was used. Samples positive for ASFV genomes in qPCR after challenge infection were thus further investigated. Primers used for detection of “ASFV-G-∆MGF” amplified a 422 bp region deleted within the MGF505-1R-Gene (primers: forward, 5 = GAGGATGATTTGCCCTTCACTCA = 3; reverse, = 5CGCCACTAGTAAACATTGTTCTATCT = 3) [14]. Amplicons were then determined by 2% agarose gel electrophoresis.

For titrations, haemadsorption test (HAT) was used under slightly modified standard procedures, as recently described [30]. Titers were calculated in accordance to method published by Spearman and Kärber [31,32].

#### 4.4.3. Antibody Detection

For the detection of ASFV-specific antibodies, two commercially available ELISA systems were used according to the manufacturer’s instructions. In the DP trials, sera were tested in the OIE-recommended p72 antibody-specific INGEZIM PPA COMPAC (Ingenasa). In the WB trial, samples were screened in the p32, p64, and p72-antibody-specific IDScreen ASF Indirect (IDVet) Kit according to the manufacturer’s instructions. The application of the IDScreen ASF Indirect kit for the WB trial was due to limitations in sample availability with the more stress-prone wild boar. INGEZIM PPA COMPAC requires serum, while plasma can be used for the IDScreen ASF Indirect assay.

For confirmatory reasons, doubtful samples were additionally tested in the indirect immunoperoxidase test (IPT) according to the standard protocols provided by the European Reference Laboratory for ASF.

## 5. Conclusions

“ASFV-G-∆MGF” is fully efficacious when administered intramuscularly, and likewise, all responders to oral immunization are protected. Passaging of the vaccine virus on a permanent cell line did not result in any alterations of characteristics, providing a basis for possible commercialization of this promising candidate. However, efficiency of oral immunization has room for improvement as only 50% of the animals seroconverted. Very limited vaccine virus replication in swine and no virus shedding were observed. Future research should now focus on safety aspects to provide a basis for evaluation by regulatory authorities of this highly promising candidate. 

## Figures and Tables

**Table 1:** Genome detection from blood samples in genome copies/5 μL.

Trial	Group	Pig #	d0	d7	d14	d21	d28	d35	d0pc	d4pc	d7pc	d10pc	d14pc	d21pc	Necr.
DP A		16	n.d.	n.d.	n.d.	n.d.	n.d.	n.d.	n.d.	n.d.	n.d.	n.d.	n.d.	n.d.	n.d.
		17	n.d.	n.d.	n.d.	n.d.	n.d.	n.d.	n.d.	n.d.	n.d.	n.d.	n.d.	n.d.	n.d.
	MGF i.m. B	18	n.d.	2.8 × 10^0^	n.d.	n.d.	n.d.	n.d.	n.d.	2.0 × 10^−1^	n.d.	n.d.	n.d.	n.d.	n.d.
		19	n.d.	n.d.	n.d.	2.0 × 10^2^	n.d.	n.d.	n.d.	n.d.	n.d.	n.d.	n.d.	n.d.	n.d.
		20	n.d.	n.d.	n.d.	n.d.	n.d.	n.d.	n.d.	n.d.	n.d.	3.7 × 10^−1^	3.2 × 10^−2^	n.d.	n.d.
		6	n.d.	n.d.	n.d.	n.d.	n.d.	n.d.	n.d.	n.d.	4.8 × 10^3^	✞			5.8 × 10^5^
		7	n.d.	n.d.	n.d.	n.d.	n.d.	n.d.	n.d.	n.d.	✞				9.5 × 10^4^
	control A	8	n.d.	n.d.	n.d.	n.d.	n.d.	n.d.	n.d.	n.d.	✞				3.5 × 10^4^
		9	n.d.	n.d.	n.d.	n.d.	n.d.	n.d.	n.d.	5.1 × 10^4^	✞				1.1 × 10^6^
		10	n.d.	n.d.	n.d.	n.d.	n.d.	n.d.	n.d.	n.d.	1.3 × 10^5^	✞			2.7 × 10^5^
DP B		21	n.d.	n.d.	n.d.	n.d.	n.d.	n.d.	n.d.	n.d.	n.d.	n.d.	n.d.	n.d.	n.d.
		22	n.d.	n.d.	n.d.	n.d.	n.d.	n.d.	n.d.	n.d.	n.d.	n.d.	n.d.	n.d.	n.d.
	MGF i.m. B	23	n.d.	n.d.	n.d.	n.d.	n.d.	n.d.	n.d.	n.d.	n.d.	n.d.	n.d.	n.d.	n.d.
		24	n.d.	n.d.	1.0 × 10^0^	n.d.	n.d.	n.d.	n.d.	n.d.	n.d.	n.d.	n.d.	n.d.	n.d.
		25	n.d.	3.5 × 10^0^	n.d.	n.d.	n.d.	n.d.	n.d.	n.d.	n.d.	n.d.	n.d.	n.d.	n.d.
	control B	42	n.d.						n.d.	n.d.	n.d.	1.1 × 10^3^	✞		7.4 × 10^4^
		43	n.d.						n.d.	n.d.	4.9 × 10^3^	✞			1.5 × 10^5^
		44	n.d.						n.d.	9.7 × 10^0^	3.7 × 10^4^	✞			1.0 × 10^5^
		45	n.d.						n.d.	4.5 × 10^4^	6.2 × 10^4^	✞			1.5 × 10^5^
WB	MGF oral	61	n.d.			9.7 × 10^1^									n.d.
		62	n.d.			n.d.									9.2 × 10^4^
		63	n.d.			3.3 × 10^1^									n.d.
		64	n.d.			n.d.									n.d.
		65	n.d.			1.2 × 10^0^									n.d.
		66	n.d.			n.d.									n.d.
		67	n.d.			n.d.									2.7 × 10^5^
		68	n.d.			n.d.									n.d.
	control WB	69							n.d.						4.4 × 10^5^
		70							n.d.						4.1 × 10^5^
		71							n.d.						1.9 × 10^5^
		72							n.d.						2.8 × 10^5^

DP: domestic pig, WB: wild boar, necr.: necropsy, ✞ animal already euthanized.

**Table 2:** Genome detection from tissue samples in genome copies/5 μL.

Trial	Group	Animal #	Lung	Spleen	Kidney	Liver	Hep. Ln.	Popl. Ln.	Mand. Ln.	Tonsil
DP A		16	n.d.	n.d.	n.d.	n.d.	n.d.	n.d.		
		17	n.d.	n.d.	n.d.	n.d.	n.d.	n.d.		
	MGF i.m. A	18	n.d.	n.d.	n.d.	n.d.	n.d.	n.d.		
		19	n.d.	n.d.	n.d.	n.d.	n.d.	n.d.		
		20	1.1 × 10^0^	n.d.	n.d.	n.d.	n.d.	8.7 × 10^1^		
		6	9.4 × 10^2^	1.3 × 10^3^	5.3 × 10^0^	1.2 × 10^3^	2.0 × 10^1^	6.4 × 10^1^		
		7	1.8 × 10^2^	7.3 × 10^2^	1.3 × 10^0^	8.8 × 10^1^	9.4 × 10^−1^	n.d.		
	control A	8	4.0 × 10^1^	1.6 × 10^3^	1.8 × 10^0^	3.3 × 10^2^	4.9 × 10^−1^	4.5 × 10^0^		
		9	1.4 × 10^3^	1.5 × 10^3^	6.7 × 10^1^	2.4 × 10^3^	6.4 × 10^2^	9.0 × 10^2^		
		10	2.1 × 10^2^	8.9 × 10^2^	6.2 × 10^0^	2.4 × 10^2^	3.8 × 10^1^	9.5 × 10^1^		
DP B		21	n.d.	n.d.	n.d.	n.d.	n.d.	n.d.		
		22	n.d.	n.d.	n.d.	n.d.	n.d.	n.d.		
	MGF i.m. B	23	n.d.	n.d.	n.d.	n.d.	n.d.	n.d.		
		24	n.d.	n.d.	n.d.	n.d.	n.d.	n.d.		
		25	n.d.	n.d.	n.d.	n.d.	n.d.	n.d.		
	control B	42	8.3 × 10^2^	9.3 × 10^2^	9.8 × 10^0^	5.3 × 10^2^	9.0 × 10^0^	3.0 × 10^0^		
		43	8.8 × 10^1^	5.0 × 10^2^	1.2 × 10^1^	6.5 × 10^2^	5.0 × 10^1^	7.0 × 10^1^		
		44	3.6 × 10^2^	6.9 × 10^2^	1.8 × 10^1^	4.8 × 10^2^	6.7 × 10^2^	3.6 × 10^2^		
		45	3.2 × 10^2^	1.1 × 10^3^	2.8 × 10^1^	2.4 × 10^3^	5.1 × 10^2^	2.1 × 10^2^		
WB	MGF oral	61	n.d.	n.d.	n.d.	n.d.	1.2 × 10^0^		2.0 × 10^0^	9.2 × 10^0^
		62	1.6 × 10^4^	3.5 × 10^4^	3.0 × 10^3^	7.7 × 10^3^	1.1 × 10^5^		4.0 × 10^3^	6.1 × 10^4^
		63	n.d.	n.d.	n.d.	n.d.	n.d.		n.d.	n.d.
		64	n.d.	1.6 × 10^−1^	n.d.	n.d.	n.d.		n.d.	n.d.
		65	n.d.	n.d.	n.d.	n.d.	n.d.		n.d.	n.d.
		66	n.d.	n.d.	2.7 × 10^−1^	n.d.	n.d.		n.d.	5.1 × 10^−1^
		67	1.0 × 10^4^	7.5 × 10^4^	2.7 × 10^3^	2.1 × 10^4^	1.1 × 10^4^		2.9 × 10^3^	1.3 × 10^4^
		68	n.d.	n.d.	n.d.	n.d.	n.d.		n.d.	n.d.
	control WB	69	4.4 × 10^4^	1.0 × 10^5^	3.4 × 10^3^	3.9 × 10^4^	4.1 × 10^4^		1.4 × 10^4^	2.4 × 10^4^
		70	1.3 × 10^4^	7.0 × 10^4^	2.4 × 10^3^	1.6 × 10^4^	2.7 × 10^4^		1.1 × 10^4^	1.9 × 10^4^
		71	6.8 × 10^3^	5.4 × 10^4^	1.1 × 10^3^	2.4 × 10^4^	1.2 × 10^4^		1.4 × 10^3^	4.7 × 10^2^
		72	1.3 × 10^4^	5.6 × 10^4^	1.6 × 10^3^	5.4 × 10^4^	1.8 × 10^4^		1.1 × 10^3^	1.9 × 10^4^

## Data Availability

All data to support the findings described in the text are included in the main text or in the Supplementary materials. Additional data are available from the corresponding author upon reasonable request.

## Supplementary Materials

The following supporting information can be downloaded at: https://www.mdpi.com/article/10.3390/pathogens11090996/s1, Table S1: back titration of viruses used for inoculations; Table S2: blocking percentages from sera; Table S3: genome copies detected in swabs by qPCR.
